# Feasibility of real-time *in vivo* ^89^Zr-DFO-labeled CAR T-cell trafficking using PET imaging

**DOI:** 10.1371/journal.pone.0223814

**Published:** 2020-01-07

**Authors:** Suk Hyun Lee, Hyunsu Soh, Jin Hwa Chung, Eun Hye Cho, Sang Ju Lee, Ji-Min Ju, Joong Hyuk Sheen, Hyori Kim, Seung Jun Oh, Sang-Jin Lee, Junho Chung, Kyungho Choi, Seog-Young Kim, Jin-Sook Ryu

**Affiliations:** 1 Department of Nuclear Medicine, Asan Medical Center, University of Ulsan College of Medicine, Seoul, Republic of Korea; 2 Department of Radiology, Division of Nuclear Medicine, Hallym University Kangnam Sacred Heart Hospital, Hallym University College of Medicine, Seoul, Republic of Korea; 3 Asan Institute for Life Sciences, Asan Medical Center, Seoul, Republic of Korea; 4 Convergence Medicine Research Center, Asan Medical Center, Seoul, Republic of Korea; 5 Research Institute, National Cancer Center, Gyeonggi-do, Republic of Korea; 6 Department of Biomedical Sciences, Seoul National University, Seoul, Republic of Korea; Monash University, AUSTRALIA

## Abstract

**Introduction:**

Chimeric antigen receptor (CAR) T-cells have been recently developed and are producing impressive outcomes in patients with hematologic malignancies. However, there is no standardized method for cell trafficking and *in vivo* CAR T-cell monitoring. We assessed the feasibility of real-time *in vivo* ^89^Zr-p-Isothiocyanatobenzyl-desferrioxamine (Df-Bz-NCS, DFO) labeled CAR T-cell trafficking using positron emission tomography (PET).

**Results:**

The ^89^Zr-DFO radiolabeling efficiency of Jurkat/CAR and human peripheral blood mononuclear cells (hPBMC)/CAR T-cells was 70%–79%, and cell radiolabeling activity was 98.1–103.6 kBq/10^6^ cells. Cell viability after radiolabeling was >95%. Cell proliferation was not significantly different during the early period after radiolabeling, compared with unlabeled cells; however, the proliferative capacity decreased over time (day 7 after labeling). IL-2 or IFN-γ secretion was not significantly different between unlabeled and labeled CAR T-cells. PET/magnetic resonance imaging in the xenograft model showed that most of the ^89^Zr-DFO-labeled Jurkat/CAR T-cells were distributed in the lung (24.4% ± 3.4%ID) and liver (22.9% ± 5.6%ID) by one hour after injection. The cells gradually migrated from the lung to the liver and spleen by day 1, and remained stable in these sites until day 7 (on day 7: lung 3.9% ± 0.3%ID, liver 36.4% ± 2.7%ID, spleen 1.4% ± 0.3%ID). No significant accumulation of labeled cells was identified in tumors. A similar pattern was observed in *ex vivo* biodistributions on day 7 (lung 3.0% ± 1.0%ID, liver 19.8% ± 2.2%ID, spleen 2.3% ± 1.7%ID). ^89^Zr-DFO-labeled hPBMC/CAR T-cells showed a similar distribution, compared with Jurkat/CAR T-cells, on serial PET images. CAR T cell distribution was cross-confirmed by flow cytometry, Alu polymerase chain reaction, and immunohistochemistry.

**Conclusion:**

Real-time *in vivo* cell trafficking is feasible using PET imaging of ^89^Zr-DFO-labeled CAR T-cells. This can be used to investigate cellular kinetics, initial *in vivo* biodistribution, and safety profiles in future CAR T-cell development.

## Introduction

Given shifting cancer treatment paradigms, chimeric antigen receptor (CAR) T-cell immunotherapy has been developed very rapidly [1,2]. CAR T-cells, which have been studied in the context of being used in immune regulatory cell therapies, harbor fusion proteins with the extracellular scFv domain of an antibody. These proteins recognize the characteristic antigen on the tumor cell surface and the intracellular costimulatory domain for T-cell activation. When CAR T-cells bind to the antigen on the surface of the tumor cell, a sequential costimulatory signal activates the T-cell and triggers the signaling pathway within the cell, thereby enabling the CAR T-cells to kill the tumor cell [3,4]. Moreover, because of their tumor cell-killing activity, CAR T-cells act as a “living drug” that can proliferate in the body. They also have significantly longer action than conventional chemotherapeutics and antibody drugs [5]. CAR T-cell therapy has been shown to have dramatic anticancer effects, particularly in clinical trials for patients with hematological malignancies, such as refractory B-cell malignancies, after standard treatment [6–8].

Despite its successful use in patients with B-cell malignancies, there is a lack of substantive understanding of the actions of CAR T-cells in the human body with respect to the following: 1) the limited effect of CAR T-cells on solid tumors; 2) the trafficking and biodistribution of CAR T-cells; and 3) the targeting efficacy of CAR T-cells that are injected into a patient’s body. To date, there are no available standardized methods for monitoring *in vivo* behaviors and targeting the efficacy of injected CAR T-cells. The most common (but limited) techniques used to identify CAR T-cells in the body are flow cytometry, biopsy/immunohistochemistry (IHC), enzyme-linked immunosorbent (ELISpot), and polymerase chain reaction (PCR) [9–12]. Unfortunately, none of these can monitor CAR T-cells within a live body. To optimize the efficacy of CAR T-cell immunotherapy, and to predict potential toxicities, it is necessary to develop a noninvasive imaging system that can enable the monitoring of CAR T-cell trafficking in a real-time manner. Image-based data provides a great deal of information concerning actual tracking, targeting patterns, real-time distributions, and *in vivo* maintenance for CAR T-cell therapies.

Additionally, the FDA Guidance for Industry: Preclinical Assessment of Investigational Cellular and Gene Therapy Products (updated 11/2013) acknowledged that the fate of investigational cell therapy, after *in vivo* administration, is important for characterizing the product’s activity and safety information. To determine the distribution of cells after administration, imaging methods such as the use of radioisotope-labeled cells, genetically engineered cells (e.g., green fluorescent protein expression), and nanoparticle-labeled cells (e.g., iron-dextran nanoparticles) are recommended. Unlike conventional drugs, cell therapies must acquire data through *in vitro* experiments to determine their pharmacological activities or any unrecognized toxicity. Therefore, animal models are generally recommended for evaluating cell therapies because basic information on the initial behavior, organ distribution, and targeting in the body after cell therapy are important. Nuclear medical imaging is a valid method that enables real-time monitoring of cells in the body.

Positron emission tomography (PET) is a method of diagnostic imaging that can evaluate metabolic activities in the body; injection of a radioactive tracer enables this nuclear medicine functional imaging technique. PET is a unique and important tool for the tracking of cells in preclinical and clinical studies [13,14]. It can be used for translational research, i.e. moving from preclinical to clinical studies, as this technology is highly sensitive and has excellent spatial resolution. There are two ways to label cells for imaging: direct and indirect labeling. This study was designed to monitor CAR T-cells using direct labeling. Direct labeling of cells immediately marks the cells with a radioisotope through covalent bond conjugation. Cell migration and distribution can be monitored immediately after cell injection. Herein, we establish a method of direct labeling for CAR T-cells. Since CAR T-cells can be manipulated *ex vivo*, it is possible to track the behavior and the distribution of small numbers of radiolabeled cells after *in vitro* labeling.

^89^Zr has a long physical half-life (78.4 hours) and is therefore suitable for tracking the behavior of CAR T-cells in the body. In previous studies, cells for imaging were directly labeled using isotopes conjugated with ^89^Zr-oxine or DFO moiety. [15–18]. Recently, Weist et al. proposed that ^89^Zr-oxine would be a clinically translatable method for real-time evaluation of cell therapies, especially that using CAR T-cells [19]. However, Bansal et al. [16] reported that the ^89^Zr-DFO labeling strategy was superior to that of ^89^Zr-oxine, with increased cell stability and viability. Based on existing preclinical applications, this study aimed to assess the feasibility of real-time trafficking of ^89^Zr-DFO-labeled CAR T-cells using PET imaging.

## Materials and methods

### Study design

**Fig 1:**
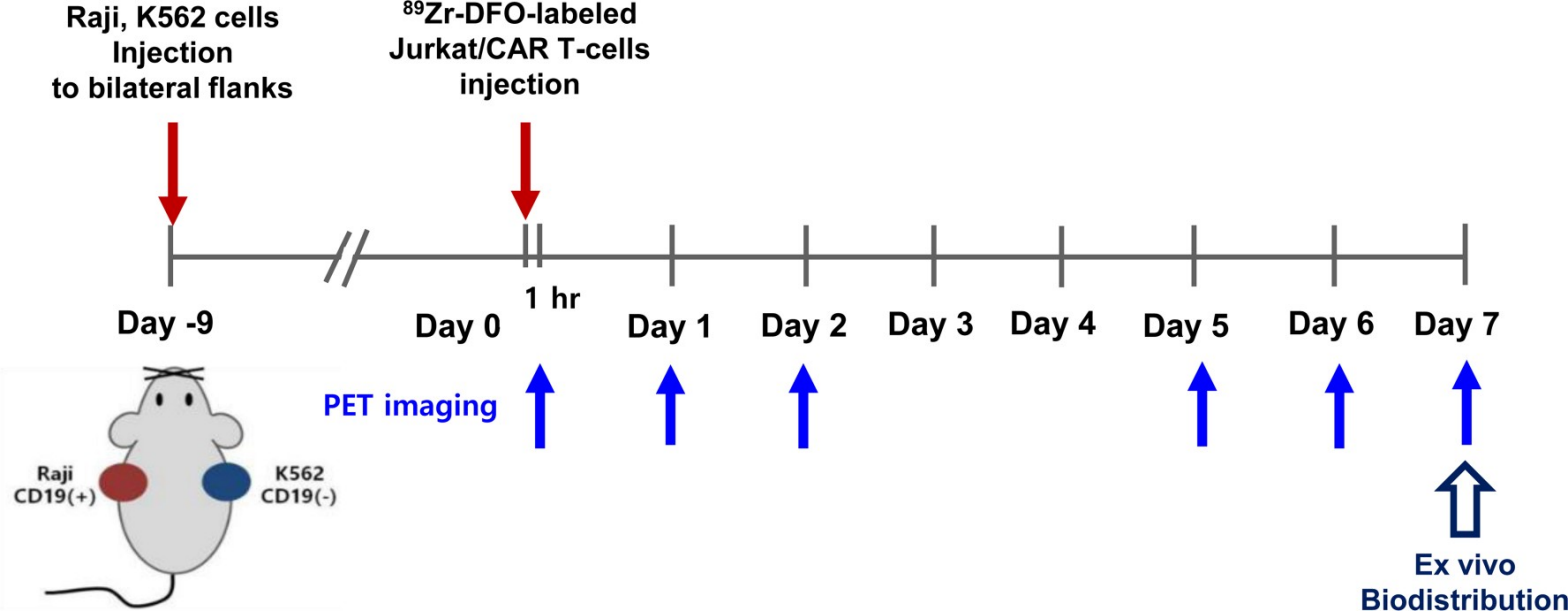
Animal study scheme.

For the exploration of CAR-T cell trafficking by PET imaging, we first performed the experiments with cultured Jurkat cells (immortalized T lymphocyte cell line), and subsequently we used human peripheral blood mononuclear cells (hPBMC). After construction of lentiviral CD19 targeting CAR vectors, we prepared CAR-expressing Jurkat (Jurkat/CAR) T-cells and CAR T-cells from hPBMC (hPBMC/CAR T-cells). All the cells were then radiolabeled with ^89^Zr-DFO. The *in vitro* viability, proliferation ability, and function of the ^89^Zr-DFO-labeled cells were evaluated. ^89^Zr-DFO-labeled Jurkat/CAR T-cells were intravenously injected into mice xenograft models with bilateral CD19 positive and negative tumors. We carried out serial imaging studies for seven days of cell trafficking *in vivo* using a PET/magnetic resonance (MR) scanner. The imaging data were compared with those of the *ex vivo* experiments performed with unlabeled Jurkat/CAR T-cells. Then, similar imaging studies were carried out using ^89^Zr-DFO-labeled hPBMC/CAR T-cells. The animal study scheme is shown in Fig 1.

The research protocol of this preclinical experimental study with animals was approved by the Institutional Animal Care and Use Committee of the Asan Institute for Life Science (registration no. 2017-12-085). Mice were maintained in accordance with the Institutional Animal Care and Use Committee guidelines of the Asan Institute for Life Science. For the hPBMC preparation, blood samples were drawn from healthy volunteer donors, who had given their written informed consent, according to the protocol approved by the Research Institute of the National Cancer Center following the Institutional Review Board (NCC2018-0030 and NCC2015-0312).

#### Cell culture and hPBMC preparation

Raji (CD19 positive cell line, ATCC), K562 (CD19 negative cell line, ATCC), and Jurkat (ATCC) were cultured in RPMI 1640 containing FBS (10%, GenDEPOT), L-glutamine (2 mmol/L, Gibco) and antibiotic-antimycotic (100x, Gibco). The hPBMCs in the blood samples were isolated by using Ficoll-Paque (GE Healthcare) and cultured as above; we additionally treated hIL-2 (200 U/ml, R&D systems).

#### Construction of a lentiviral vector containing CD19-specific CAR

**Fig 2:**
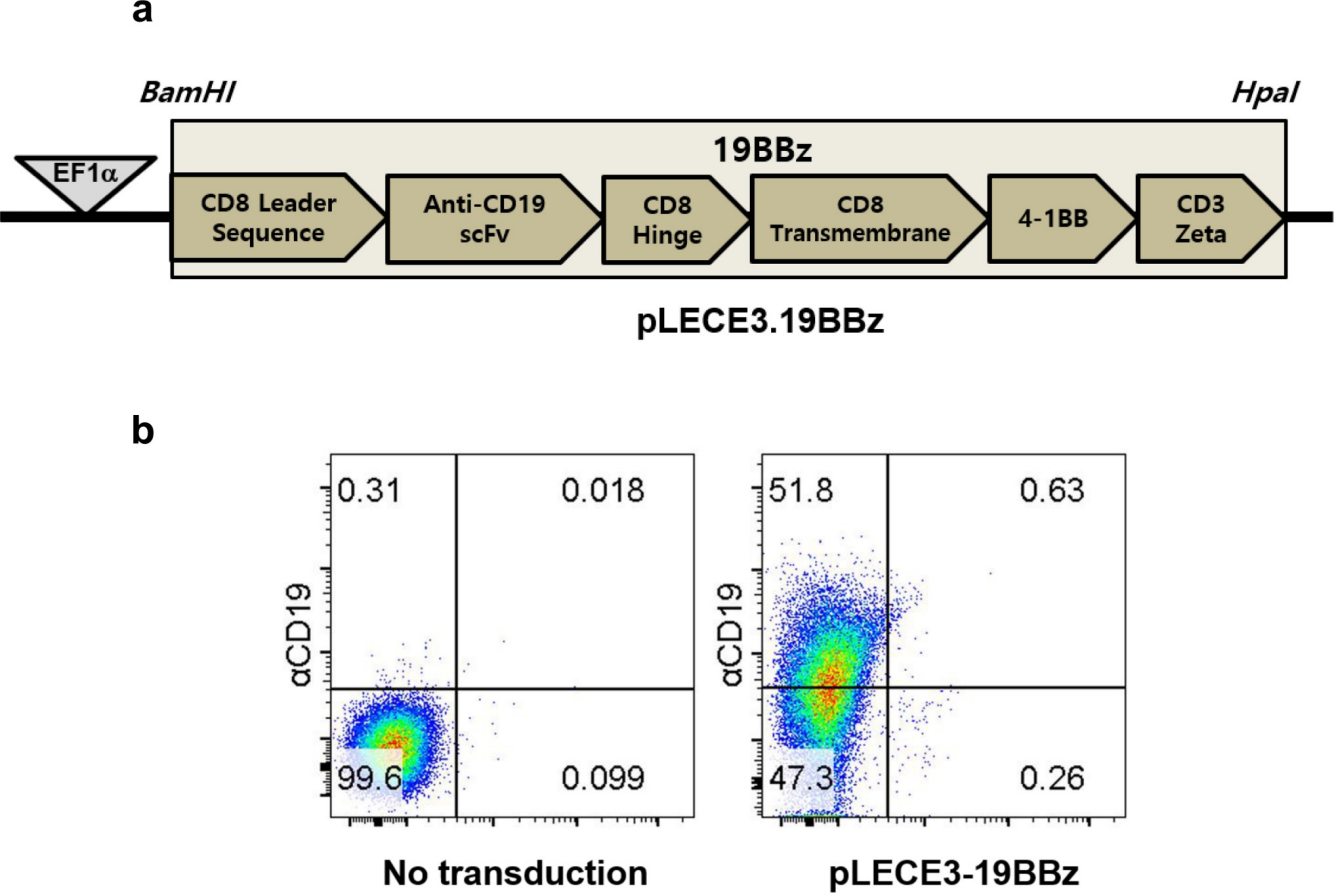
Construction of Jurkat/CAR T-cells specific to CD19. **a** DNA map of pLECE3-19BBz lentivirus vector. **b** CAR expression on Jurkat T-cells. Jurkat T-cells were either left alone (left) or transduced with pLECE-19BBz lentiviruses (right).

To construct a lentiviral vector encoding CAR specific to CD19, we generated the EF1α promoter-driven lentiviral expression vector, pLECE3. The pLECE3 was constructed by replacing the U6 promoter of pLentiLox3.7 with the EF1α promoter, together with a few additional cloning sites. The 19BBz consists of an anti-CD19 scFv, a CD8 Hinge, a CD8 transmembrane, a 4-1BB, and a CD3 ζ domain, which are identical with the Novartis Kymriah product. DNA products of 19BBz domain were amplified by PCR (19BBz; 5’-GATCCgccaccATGGCCTTA CCAGTGA-3’ and 5’-GTTAACttaGCGAGGGGGCAGGGCCTGCAT-3’). The PCR product was then subcloned into a pGEM-T-easy vector (Promega, USA). The 19BBz was inserted into the pLECE3 at the *BamHI/HpaI* site, under the EF1a promoter (Fig 2A).

#### Transfection and lentivirus packaging

The lentiviral vectors were transfected into the 293T cells with Lipofectamine 3000® (Thermo Fisher Scientific), following the manufacturer’s protocol. Briefly, 7 × 10^6^ 293T cells (ATCC) were plated in 150 mm petri dishes. The cells were treated with a lipofectamine reagent, viral vectors, and packaging DNA. For high-titer lentiviral purification, cellular supernatants were collected and filtered through a 45 μm pore filter unit (Sartorius AG, Germany) at 48 hours post transfection, and purified using ultracentrifugation.

#### Transduction of lentivirus into Jurkat T-cells and hPBMCs

We plated 4 × 10^5^ Jurkat T-cells (ATCC, TIB-152) one day before transduction. The next day, the cells were infected with the lentiviruses (MOI 1000), with polybrene (8 μl/ml, Merk, Germany) added to increase the efficiency of transduction. Then, a 90-minute spin infection was performed (800 × g). We sorted transduced Jurkat T-cells that expressed 19BBz 72 hours later using FACS Aria (BD Biosciences, USA). Briefly, the biotin-SP-conjugated goat antimouse IgG F(ab')2 fragment-specific antibody was used as a primary antibody to capture the CAR, and the PerCP/Cy5.5 Streptavidin antibody (Biolegend, USA) was used as a secondary antibody. The CD19 expression levels are shown in Fig 2B. The sorted cells were cultured again to establish stable Jurkat/CAR T-cell lines. On the other hand, CD19 hPBMC CAR T-cells were generated by transduction of hPBMCs with lentivirus prepared as described elsewhere [7].

#### Synthesis of the ^89^Zr-DFO complex for cell labeling

^89^ZrCl_4_ was prepared from the ^89^Zr-oxalate (Perkin Elmer). ^89^Zr-DFO complex was synthesized using 2.8 nmol of DFO and 62.9 ± 29.6 MBq of ^89^ZrCl_4_ and analyzed with a slight modification of the previously reported method [16]. We only changed the reagent of neutralization from KOH to NaOH, and the incubation time from one hour to overnight, to increase the chelation efficiency to over 90%.

#### Labeling of CAR T-cells with ^89^Zr-DFO

Cell labeling with ^89^Zr-DFO was performed with a slight modification of the previously described method [15]. Briefly, the Jurkat/CAR T-cells were counted, harvested and washed once with HBSS buffer (pH 7.5). Then, 185 kBq/100 μl HBSS buffer of ^89^Zr-DFO was added to each glass tube with 5 × 10^6^ cells in 500 μl HBSS buffer and incubated in a thermomixer at 37°C for 30 minutes (Eppendorf, Germany) with gentle shaking. After incubation, we added 1 ml of cold HBSS buffer, and the solution was centrifuged at 1200 rpm for five minutes at 4°C in order to separate the supernatant. The supernatant was collected in a new tube, and we repeated this washing process three times. The final cell labeling efficiency was calculated as follows: labeling efficiency (%) = [cell activity (cpm)/ {cell activity (cpm) + supernatant activity (cpm)}] × 100. CAR T-cells from the hPBMC were labeled using the same procedure as with the Jurkat /CAR T-cells, but ^89^Zr-DFO 74 kBq /100 μl HBSS buffer was added to each tube.

#### Cell viability and proliferative activity of ^89^Zr-DFO-labeled cells

To investigate the cell viability and proliferation rates, ^89^Zr-DFO-labeled and unlabeled cells were seeded, 2 × 10^4^ ~ 5 × 10^5^ cells/well in six well plates containing 5 ml of culture medium. After seeding, the cell viability was assessed using trypan blue exclusion assay at one hour, one day, three days, and seven days. Simultaneously, the cell proliferation rate was compared with that of the unlabeled cells. The seeded cells were maintained at 37°C in a 5% CO_2_ incubator. The unlabeled cells served as controls.

#### Function test for ^89^Zr-DFO-labeled cells

To determine the function of the ^89^Zr-DFO-labeled Jurkat/CAR T-cells, we evaluated the target cell-specific cytokine IL-2 production ability with CD19 positive Raji cells (Burkitt lymphoma, ATCC, CCL-86) and CD19 negative K562 cells (chronic myelogenous leukemia, ATCC, CCL-243) as target cells. We then compared the results from the ^89^Zr-DFO-labeled cells with the results obtained from the unlabeled Jurkat/CAR T-cells. For IL-2 secretion assay, ^89^Zr- DFO-labeled cells were seeded, 4 × 10^4^ cells/well in six well plates, and were cultured for 12 or 36 hours at a 4:1 ratio [effector cells: target cells (Raji or K562 cells)]. Human IL-2 ELISA assay (RayBiotech, GA, USA) was performed according to the manufacturer’s instructions. To observe the function of the ^89^Zr-DFO-labeled hPBMC CAR T-cells, we additionally performed IFN-γ release assay (RayBiotech, GA, USA) according to the manufacturer’s instructions.

#### Animal model establishment

To develop a mouse xenograft model, immunodeficient NSG mice (female, 5–6 weeks old, 20–22 g) from Jackson Laboratory (USA) were used. To compare tumor specific targeting of CAR T-cells, we used two cell lines, Raji and K562, which presented different CD19 protein expression (S2 File). 5 × 10^6^ of the Raji cells were injected into the left flank, and 5 × 10^5^ of the K562 cells were injected into the right flank, of the same mouse. The mice were maintained in accordance with the Institutional Animal Care and Use Committee guidelines of the Asan Institute for Life Science. Over the following five to seven days we selected six mice out of 12 for PET/MR imaging if the tumors reached a volume of 50 to 100 mm^3^. Mice with tumor growth failure, or inappropriate tumor size, were excluded. Tumor volume was measured three times per week and calculated as length × width × height × π/6 (mm^3^).

#### Animal PET-MR imaging and image analysis

PET-MRI fusion imaging was performed using nanoScan PET/MRI (1T, Mediso, Hungary). After careful review of previous studies [16,18,20], we obtained the images through the following process. ^89^Zr-DFO-labeled Jurkat/CAR T-cells (n, median: 4.1 × 10^6^, range: 3.1–5.4 × 10^6^; radioactivity, median: 907 kBq, range: 481–1221 kBq) were slowly injected intravenously through a tail vein of each mouse (n = 4) using a 26 G syringe. Before PET image acquisition, the mice were kept under anesthesia (1.5% isoflurane in 100% O_2_ gas). Imaging was performed on day 0 (one hour after the injection) and again on days 1, 2, 5, 6 and 7. The T1 weighted with gradient-echo three-dimensional (3D) sequence (TR = 25 ms, TEeff = 3.4, FOV = 64 mm, matrix = 128 × 128) MR images were acquired, followed by static PET images for ten minutes (days 0 and 1), 20 minutes (day 2), or 30 minutes (days 5, 6 and 7) in a 1:5 coincidence mode and a single field of view within the MRI range. Body temperature was controlled by heated air directed onto the animal bedding (Multicell, Mediso, Hungry), and a pressure-sensitive pad was used for respiratory triggering. In addition, we also obtained PET/MR images of the mice after injecting the ^89^Zr-DFO-labeled hPBMC CAR T-cells (n = 2) through a similar process, as above.

PET images were reconstructed using Tera-Tomo 3D, in full detector mode: with all the corrections on, high regularization and eight iterations. A 3D volume of interest (VOI) was applied to organs and tumors on the reconstructed PET and MR images using the InterView Fusion software package (Mediso, Hungary) and quantitative analysis procedures. Then, percentage of injected doses (%ID; percentage of radioactivity in each organ to injected radioactivity) were calculated. VOIs, with a fixed 2 mm diameter sphere, were also drawn for the tumors (Raji and K562), brains, hearts (left ventricles), lungs, livers, kidneys, spleens, and bones (femurs), and they were analyzed using the following formula: standardized uptake value (SUV) = (radioactivity in the VOI with the unit of Bq/cc × body weight) divided by injected radioactivity.

#### *Ex vivo* biodistribution study

Immediately after the PET-MR image acquisition on day 7, the ^89^Zr-DFO-labeled Jurkat/CAR T-cell injected mice (n = 4) were sacrificed by cardiac puncture under anesthesia, to reduce pain without affecting the experimental results. Their organs (brain, heart, lung, liver, spleen, kidney, stomach, intestine, bone, muscle, etc.) and tumors were excised, weighed, and counted using a gamma-counter for five minutes. The %ID values were obtained, after normalizing to the weight of each organ.

#### Flow cytometry analysis

To validate the biodistribution of Jurkat/CAR T-cells after injection, we performed *ex vivo* immunostaining of organs. The mouse organs (liver, spleen) were harvested on day 3 after injection of unlabeled Jurkat/CAR T-cells (2 × 10^7^/200 μl) into the NSG mice with tumors, via their tail veins (n = 2). The tissues were ground using a gentleMACS^™^ Dissociator (Miltenyi Biotec, Germany) in accordance with the supplier’s protocol. A mouse cell depletion kit (Miltenyi Biotec, Germany) was used to separate the Jurkat/CAR T-cells from the mouse cells. After counting the live cells, the human T-cell isolation kit (Miltenyi Biotec, Germany) was used, according to the supplier’s protocol. Red blood cells were removed using an RBC lysing buffer (Sigma Aldrich, MO) for one minute, followed by washing and re-suspension in 1x HBSS containing 1% FBS. The separated cells were used with PE-conjugated anti-CD3. The analysis staining process was the same as that used *in vitro*. Data were acquired from the stained cells using BD FACS CantoII flow cytometry (BD Biosciences). The results were evaluated with FlowJo software (Treestar Inc., Ashland, OR).

#### Preparation of genomic DNA and Alu PCR analysis

To validate the biodistribution of Jurkat/CAR T-cells after injection, we also performed PCR analysis of *ex vivo* tissue (n = 3). At three days after injection of 2 × 10^7^ of Jurkat/CAR T-cells into the mice through their tail veins, the mice were sacrificed and brain, heart, lung, liver, spleen, and kidney samples were collected. For the Alu PCR assay to detect injected human cells, genomic DNA was extracted from the tissue samples using the QIAamp® (Qiagen, Germany) according to the manufacturer’s protocol. The primers used in this study were as follows: for human Alu, 5’- CACCTGTAATCCCAGCACTTT-3’ (forward primer) and 5’-CCCAGGCTGGAGTGCAGT-3’ (reverse primer). Real-time PCR was performed using SYBR® Green Realtime PCR Master Mix (TOYOBO, Japan) and the ABI 7500 Fast Real-Time PCR System (Applied Biosystems, CA, USA), according to the manufacturers’ instructions. The PCR experimental conditions were: 95°C for ten minutes, followed by 40 cycles at 95°C for 15 seconds and 60°C for one minute. This was followed by melting curve cycles at 95°C for 15 seconds, at 60°C for one minute, and finally at 95°C for 15 seconds. We used concentrations that were one, 5, and 25 dilutions based on 10^6^ cells of Jurkat/CAR T-cells expressing CD19 as a standard. The results indicated the amount of Alu expression, based on the standard.

#### Immunohistochemistry analysis

IHC analysis was performed as previously described [20]. The mice were sacrificed on day 3 (n = 3) or day 7 (n = 2) after Jurkat/CAR or hPBMC CAR T-cells were injected into the tumor-bearing mice. With the day 3 group, after injection of Jurkat/CAR T-cells, the liver, spleen, and tumors were harvested and fixed within paraffin blocks for IHC staining. The slides were stained with an anti-CD3 antibody (Abcam, UK) with the Dako REAL^™^ EnVision^™^ Detection System (Agilent Technologies, Inc., CA, USA) and were counter-stained with hematoxylin.

### Statistical analysis

Data are shown as mean ± SD unless otherwise stated. A value of p < 0.05 was considered to be statistically significant. The Kruskal-Wallis test was used to determine the differences between time points after ^89^Zr-DFO labeling, and the Mann-Whitney test was used to determine the differences between ^89^Zr-DFO-labeled and unlabeled cells. Statistical analyzes were performed by GraphPad Prism (GraphPad Software, CA, USA).

## Results

### Labeling efficiency, viability and proliferative ability of ^89^Zr-DFO-labeled Jurkat/CAR T-cells and CAR T-cells

**Fig 3:**
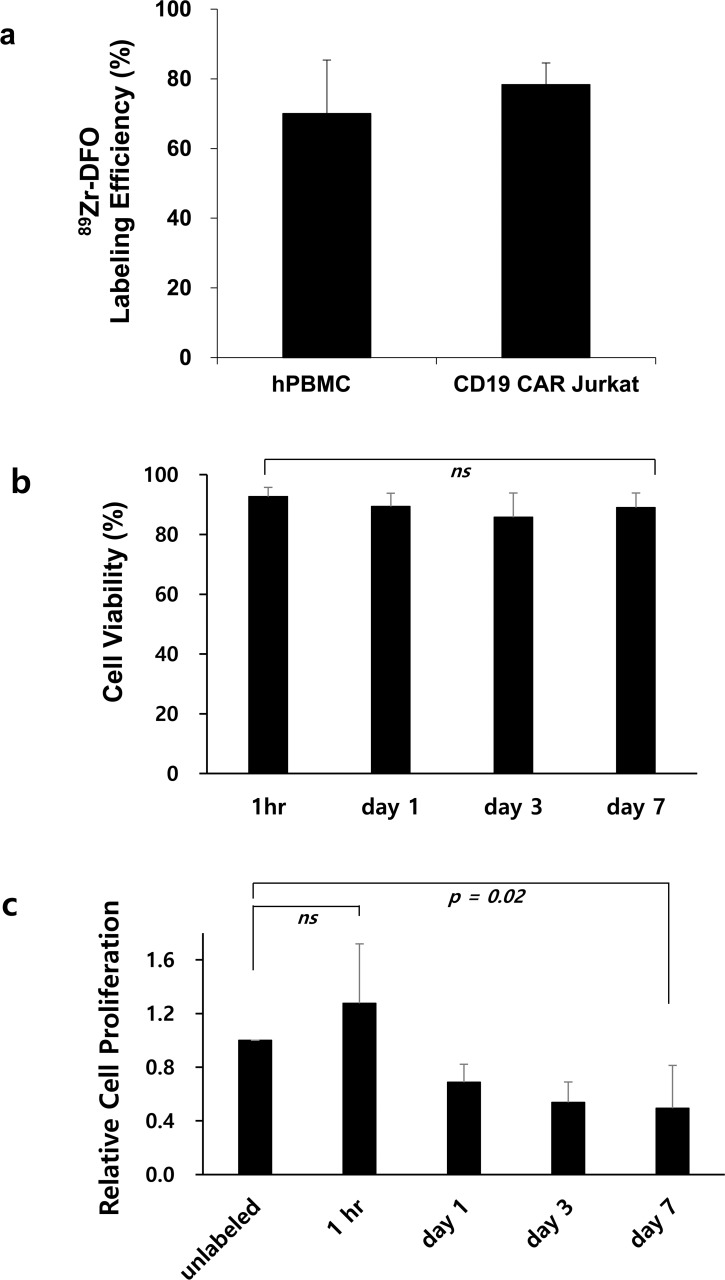
^89^Zr-DFO-labeled CAR T-cells labeling efficiency, cell viability and relative cell proliferation. **a** hPBMC CAR T-cells labeling efficiency after ^89^Zr-DFO labeling. **b** The cell viability was measured up to seven days after cell labeling **c** Relative cell proliferation of the labeled CAR T-cells was compared to that of the unlabeled. Data are representative of at least three independent experiments. All data are expressed as the mean and standard deviation.

The ^89^Zr-DFO labeling efficiency of Jurkat/CAR T-cells was 72.8% ± 11.0% at 185 kBq (Fig 3A). Cell viability after ^89^Zr-DFO labeling was 95.2% ± 1.2%, similar to the levels obtained before labeling. The concentration of radioactivity for the Jurkat/CAR T-cells was 103.6 kBq/10^6^ cells. The labeling of hPBMC CAR T-cells proceeded to 74 kBq. The ^89^Zr-DFO labeling efficiency was similar to that of the Jurkat/CAR T-cells (Fig 3A). The ^89^Zr-DFO-labeled hPBMC CAR T-cells showed a radioactivity concentration of 98.1 kBq/10^6^ cells.

We checked cell viability and cell proliferative ability at one hour, one day, three days and seven days after ^89^Zr-DFO cell labeling. There was no statistically significant difference in cell viability, although viability decreased over time after cell labeling (p = 0.24, 1 hour: 92.6% ± 3.1%, day 1: 89.4% ± 4.4%, day 3: 85.7% ± 8.1%, day 7: 89.0% ± 4.9%, respectively) (Fig 3B). However, the ^89^Zr-DFO-labeled cells showed decreased proliferative ability in a time-dependent manner (Fig 3C). At one hour after cell labeling, cell proliferation ability was not significantly changed, compared with that of the unlabeled cells (*p* = 0.25). However, cell proliferation significantly decreased over time (1 hour: 1.28 ± 0.44, day 1: 0.69 ± 0.13, day 3: 0.54 ± 0.15, and day 7: 0.49 ± 0.32, respectively).

### Functional test for ^89^Zr-DFO-labeled Jurkat/CAR T-cells or CAR T-cells

**Fig 4:**
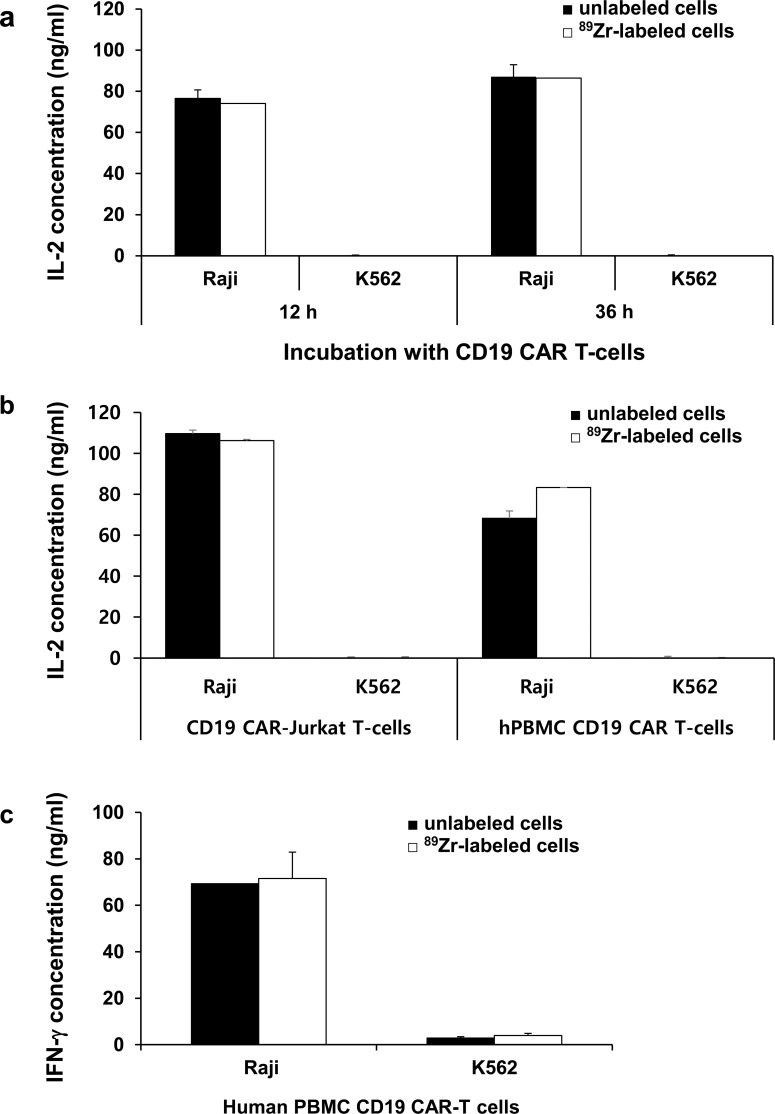
Cell function test after ^89^Zr-DFO-labeled Jurkat/CAR T-cells and hPBMC CAR T-cells. **a** Function test by IL-2 production of ^89^Zr-DFO-labeled Jurkat/CAR T-cells after incubation with Raji (CD19 positive) or K562 (CD19 negative) cells. Incubation time was 12 hours or 36 hours, and unlabeled cells were used in the experiment as a control group. **b** Function test by IL-2 production of ^89^Zr-DFO-labeled Jurkat/CAR T-cells or hPBMC CAR T-cells after incubation with Raji or K562 cells. **c** IFN-γ test of ^89^Zr-DFO-labeled hPBMC CAR T-cells with Raji or K562 cells. Data are representative of at least two independent experiments. All data are expressed as means and standard deviations.

CAR T-cell function was assessed by target cell-specific cytokine IL-2 production ability at 12 and 36 hours after cell labeling. Both ^89^Zr-DFO-labeled and unlabeled Jurkat/CAR T-cells induced IL-2 release in the positive target cells (Raji) at similar levels (74.1 vs 76.5 ng/ml at 12 hours, *p* = 0.99; 86.4 vs 87.1 ng/ml at 36 hours, *p* = 0.85) (Fig 4A). IL-2 was not released in the negative target cells (K562), which did not react with the Jurkat/CAR T-cells.

The IL-2 secretion of hPBMC ^89^Zr-DFO-labeled CAR T-cells was also similar to that observed of the unlabeled cells (Fig 4B). When hPBMC CAR T-cells were added to Raji cells expressing the targeted CD19 cells, the secretion of IFN-γ involved in cell cytotoxicity was maintained after ^89^Zr-DFO labeling (69.3 vs 71.6 ng/ml, *p* = 0.67) (Fig 4C).

### Trafficking of ^89^Zr-DFO-labeled Jurkat/CAR T-cells with PET/MR imaging

**Fig 5:**
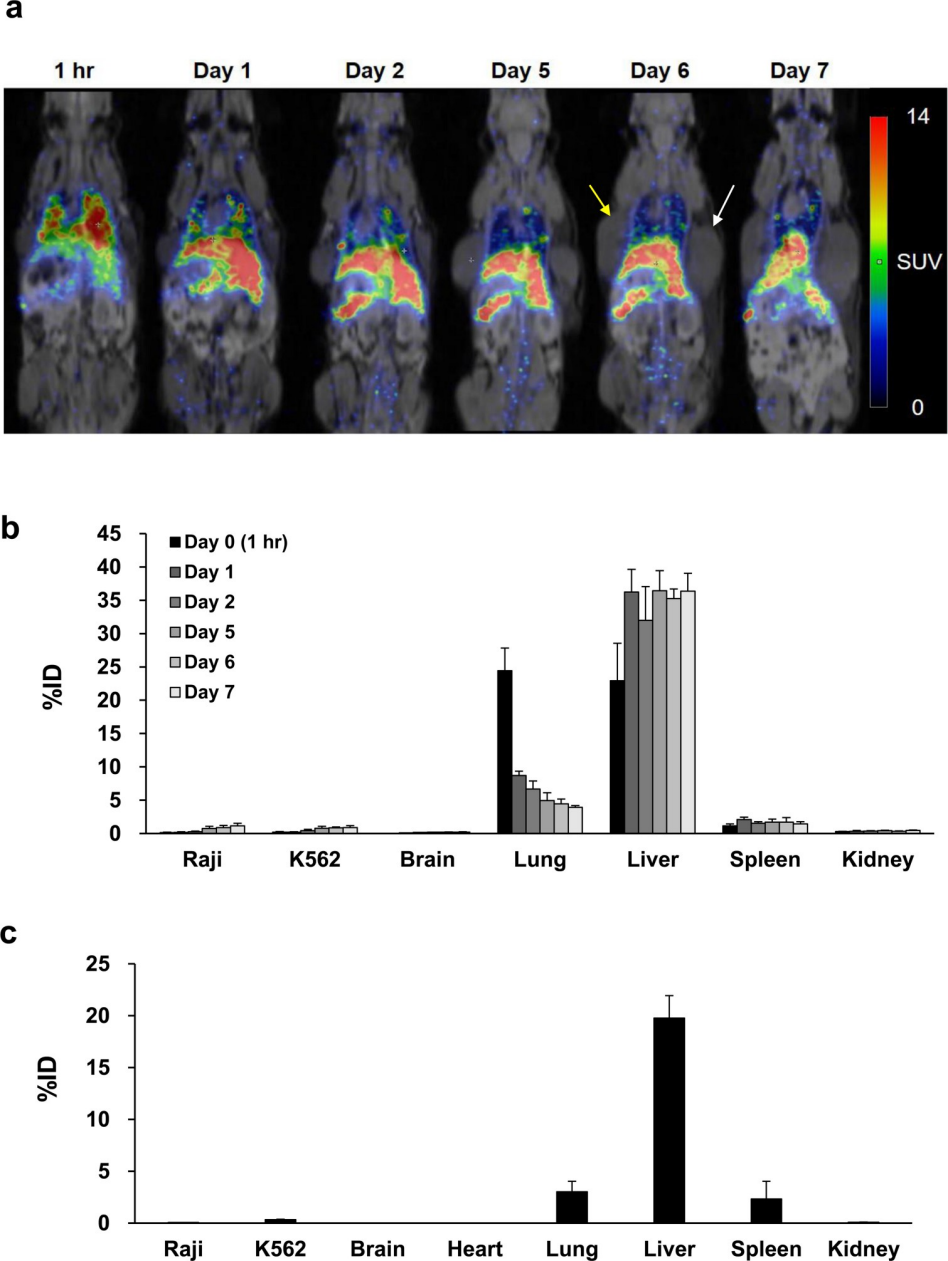
Serial ^89^Zr-DFO-labeled Jurkat/CAR T-cells animal PET/MR images and analysis of biodistribution. **a** PET images of the whole-body distribution of intravenously injected ^89^Zr-DFO-labeled CAR T-cells in NSG mouse xenograft over the following seven days. Yellow arrow and white arrow represent Raji tumor and K562 tumor, respectively. **b** The *in vivo* organ distribution of ^89^Zr-DFO-labeled Jurkat/CAR T-cells measured by PET imaging over the following seven days. **c** The distribution of *ex vivo* organ measured by model sacrifice after acquisition of the last PET imaging. (n = 4).

After injection of ^89^Zr-DFO-labeled Jurkat/CAR T-cells through the tail veins of the mice, we used PET/MR imaging for noninvasive real-time tracking of the injected cells *in vivo*. The Jurkat/CAR T-cells were initially located in the lungs, then redistributed to the liver and spleen (Fig 5A). In more detail, the injected ^89^Zr-DFO-labeled Jurkat/CAR T-cells were found mainly in the lung (24.4% ± 3.4%ID) and liver (22.9% ± 5.6%ID) during the first hour. Over time, the CAR T-cells gradually migrated from the lungs and accumulated mainly in the liver, with some in the spleen (Fig 5B). However, radioactivity accumulation was not evident in either CD19-positive Raji or CD19-negative K562 tumors. The detailed quantitative values of the PET images, analyzed by SUV, are shown in Table 1. Immediately after PET/MR imaging on day 7, the mice were sacrificed; their organs and tumors were then isolated to measure the radioactivity of injected cells *ex vivo*. The biodistribution data measured *ex vivo* on day 7 showed similar patterns of distribution to those assessed by the PET images, as shown in Fig 5C and Table 2.

**Table 1:** Quantitative analyses of ^89^Zr-DFO-labeled Jurkat/CAR T-cells on PET images with SUV (mean ± SD, n = 4).

Tissues	Day 0 (1 hour)	Day 1	Day 2	Day 5	Day 6	Day 7
Raji	0.29 ± 0.04	0.26 ± 0.04	0.25 ± 0.05	0.21 ± 0.01	0.19 ± 0.00	0.20 ± 0.03
K562	0.51 ± 0.18	0.43 ± 0.09	0.53 ± 0.22	0.42 ± 0.11	0.37 ± 0.04	0.35 ± 0.10
Brain	0.05 ± 0.01	0.09 ± 0.01	0.10 ± 0.01	0.12 ± 0.02	0.13 ± 0.02	0.15 ± 0.02
Heart	1.19 ± 1.16	0.39 ± 0.03	0.36 ± 0.07	0.23 ± 0.04	0.26 ± 0.07	0.24 ± 0.07
Lung	11.72 ± 1.84	4.31 ± 0.67	3.50 ± 0.40	2.63 ± 0.49	2.67 ± 0.60	2.22 ± 0.46
Liver	7.02 ± 0.69	9.40 ± 0.60	10.24 ± 0.86	10.16 ± 0.69	10.28 ± 0.83	9.54 ± 0.51
Spleen	5.29 ± 0.49	7.71 ± 0.99	9.02 ± 2.78	10.59 ± 0.89	9.81 ± 1.22	10.22 ± 0.59
Kidney	0.36 ± 0.04	0.35 ± 0.04	0.39 ± 0.05	0.49 ± 0.08	0.42 ± 0.04	0.51 ± 0.09
Bone	0.23 ± 0.04	0.79 ± 0.23	0.81 ± 0.36	0.98 ± 0.19	0.88 ± 0.17	0.81 ± 0.16

**Table 2:** Quantitative analyses of the *ex vivo* biodistribution of ^89^Zr-DFO-labeled Jurkat/CAR T-cells study on day 7 (%ID/g; mean ± SD, n = 4).

Tissues	%ID/g
Raji	0.09 ± 0.01
K562	0.08 ± 0.01
Blood	0.04 ± 0.01
Brain	0.01 ± 0.01
Heart	0.12 ± 0.05
Lung	24.01 ± 8.05
Liver	27.01 ± 2.96
Spleen	118.65 ± 87.60
Kidney	0.30 ± 0.10
Stomach	0.06 ± 0.01
S-intestine	0.04 ± 0.01
L-intestine	0.03 ± 0.01
Ovary	0.26 ± 0.25
T-spine	1.13 ± 0.17
Femur	2.31 ± 1.06
Muscle	0.04 ± 0.01

**Fig 6:**
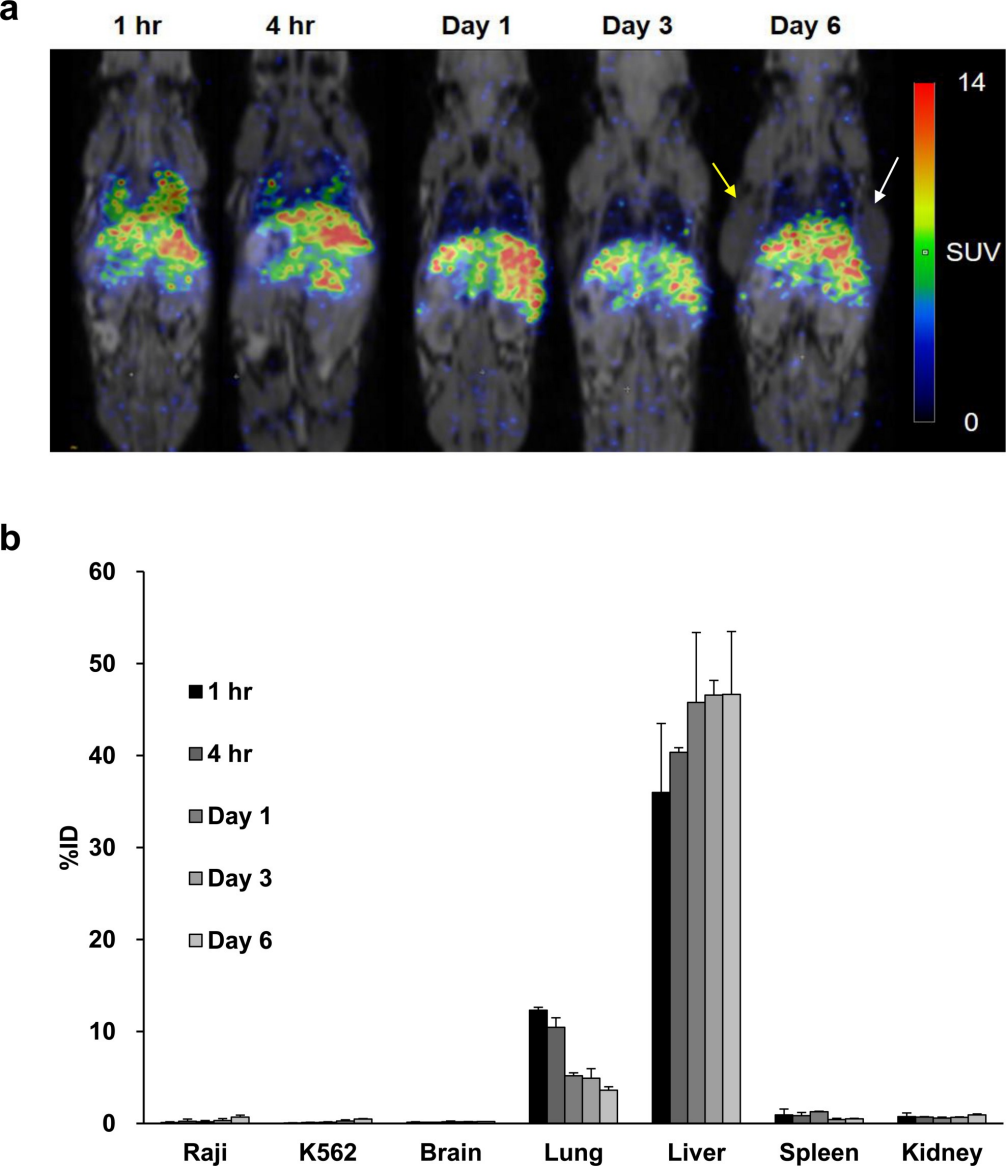
Serial ^89^Zr-DFO-labeled hPBMC CAR T-cells animal PET/MR images and analysis of biodistribution. **a** PET images of whole-body distribution for intravenously injected ^89^Zr-DFO-labeled hPBMC CAR T-cells in NSG mouse xenograft over the following six days. Yellow arrow and white arrow represent Raji tumor and K562 tumor, respectively. **b** The distribution in *in vivo* organs measured by ^89^Zr-DFO PET imaging over the following six days (n = 2).

PET/MR imaging with ^89^Zr-DFO-labeled CAR T-cells from hPBMC also showed similar biodistribution of cells after tail vein injection. We did not observe increased radioactivity in the tumors, which would have suggested CAR T-cell homing (Fig 6). The detailed quantitative values analyzed by SUV are shown in Table 3.

**Table 3:** Quantitative analyses of ^89^Zr-DFO-labeled hPBMC CAR T-cells on PET images with SUV (mean ± SD, n = 2).

Tissues	Day 0 (1 hour)	Day 0 (4 hours)	Day 1	Day 3	Day 6
Raji	0.57 ± 0.12	0.74 ± 0.45	0.55 ± 0.16	0.34 ± 0.12	0.47 ± 0.23
K562	0.14 ± 0.02	0.20 ± 0.11	0.20 ± 0.03	0.18 ± 0.00	0.29 ± 0.07
Brain	0.10 ± 0.01	0.12 ± 0.02	0.12 ± 0.04	0.15 ± 0.04	0.51 ± 0.43
Heart	0.74 ± 0.37	0.56 ± 0.20	0.27 ± 0.14	0.32 ± 0.04	0.32 ± 0.02
Lung	5.79 ± 0.25	3.71 ± 0.03	2.18 ± 0.33	2.15 ± 0.20	1.99 ± 0.63
Liver	9.07 ± 0.04	10.51 ± 1.23	10.20 ± 0.31	10.38 ± 0.16	8.81 ± 3.19
Spleen	4.34 ± 0.32	7.29 ± 0.88	6.12 ± 0.37	3.78 ± 0.27	4.30 ± 1.09
Kidney	0.81 ± 0.50	0.62 ± 0.15	0.43 ± 0.09	0.54 ± 0.00	1.22 ± 0.61
Bone	0.30 ± 0.15	0.49 ± 0.14	0.64 ± 0.35	0.48 ± 0.40	1.19 ± 0.71

### Flow cytometry, Alu PCR and immunohistochemistry for the *ex vivo* organ study

**Fig 7:**
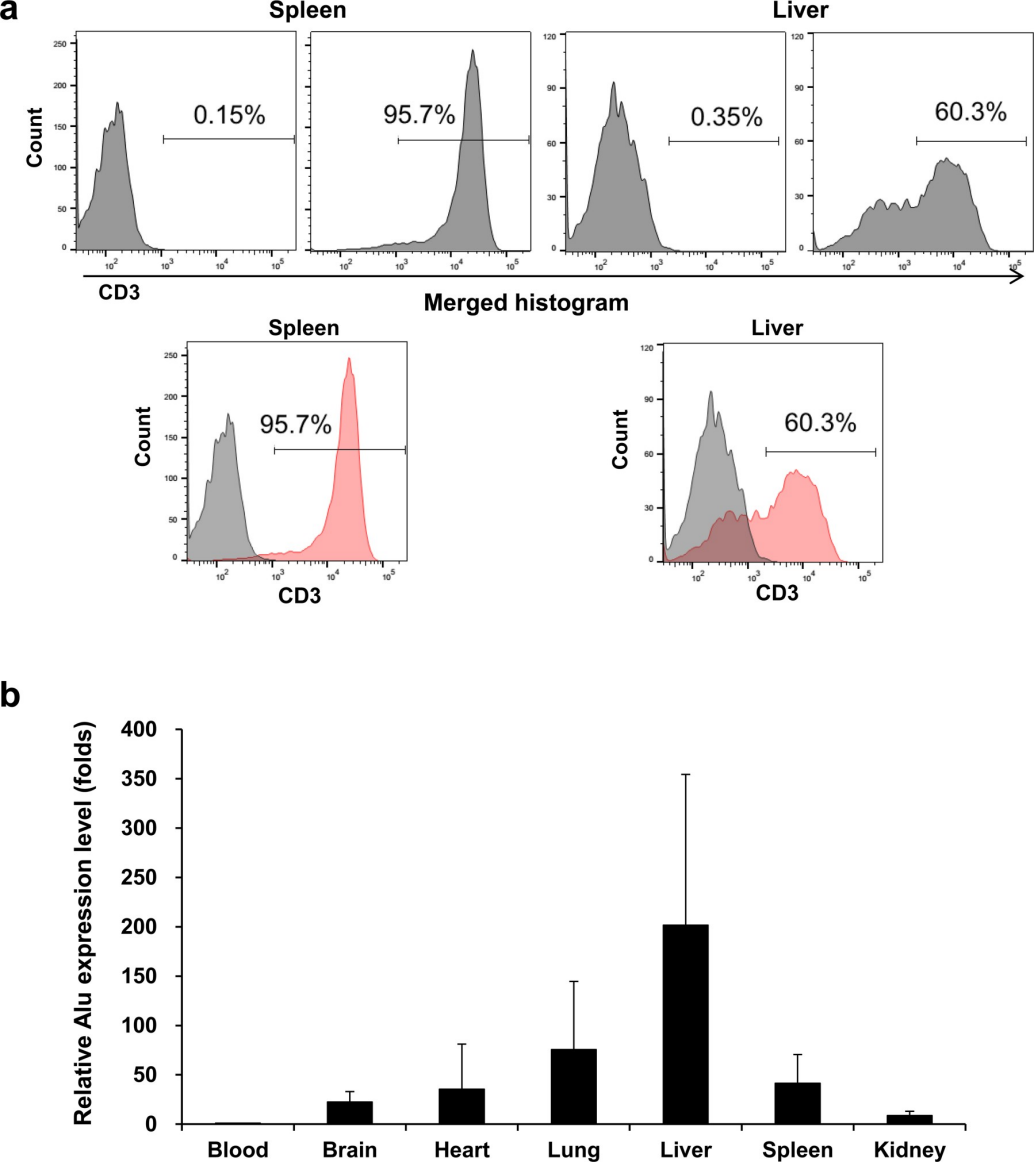
FACS staining and Alu PCR in mouse organs. **a** Post sacrificing the mice on day 3 after Jurkat/CAR T-cells injection, graphs of FACS staining for liver and spleen tissues of mice were plotted against the control group. **b** Alu PCR analysis data of mouse blood, brain, heart, lung, liver, spleen, kidney and gut tissues obtained from sacrifice three days after Jurkat/CAR T-cells injection.

We evaluated the biodistribution of unlabeled Jurkat/CAR T-cells after injection using nonimaging methods, for comparison with the evaluation using the imaging method. For flow cytometry, the liver and spleen tissue samples from mice were separated into single cells. After the initial separation, an average number of 3.05 × 10^7^ and 1.67 × 10^6^ cells per mouse were harvested. After depletion of mouse liver cells, an average of 4.74 × 10^4^ cells was obtained after using a human T-cell isolation kit. In contrast with the negative control stained with isoform antibody, CD3 expressing CAR T-cells were distributed in 95.7% of the spleen and 60.3% of the liver (Fig 7A).

Organ distributions of Jurkat/CAR T-cells were confirmed through Alu PCR. The blood, heart, lung, liver, spleen and kidney were sampled, and CD19 CAR T-cells were distributed in all six organs. The relative Alu expression was determined by measuring the blood, and the fold was as follows: brain (15.1 ± 5.8), heart (6.2 ± 7.2), lung (38.5 ± 34.8), liver (212.2 ± 225.4), spleen (70.3 ± 9.4), kidney (9.4 ± 6.4) and gut (17.5 ± 8.9) (Fig 7B).

**Fig 8:**
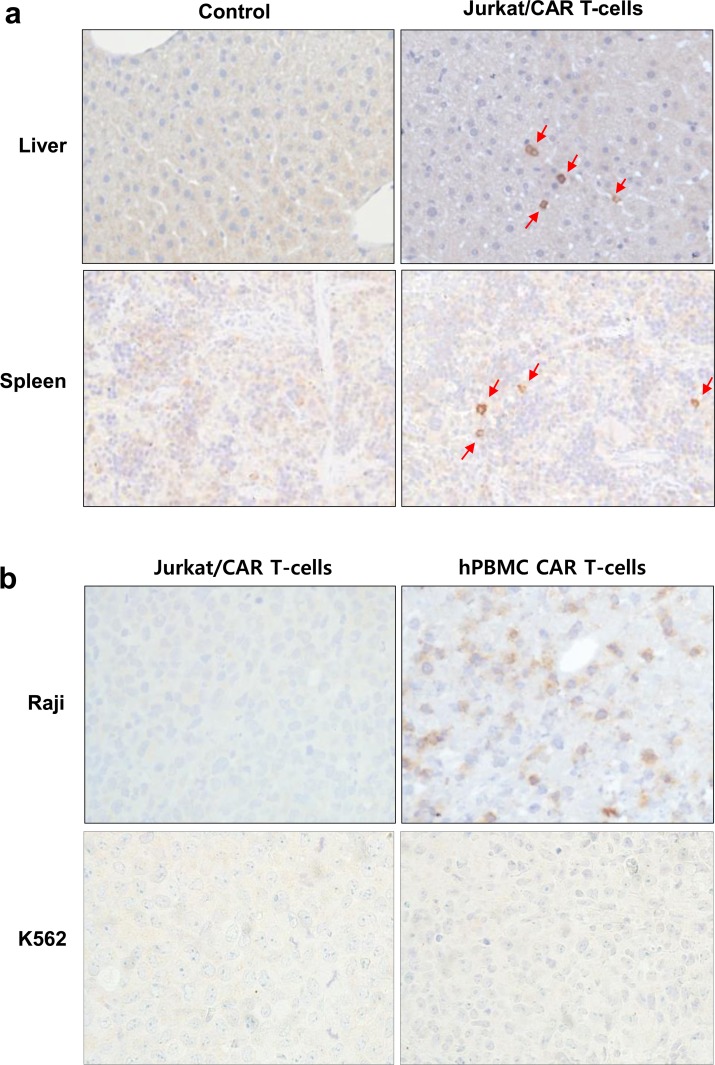
IHC staining in mouse organs. **a** IHC staining with CD3 antibody demonstrates increased staining in liver and spleen tissue after Jurkat/CAR T-cells were injected into a mouse, compared with control mouse spleen. Red arrows show CD3 targeting T-cells in IHC staining. **b** IHC staining with CD3 antibody in Raji and K562 tumor tissues on day 7 after Jurkat/CAR or hPBMC CAR T-cells injection.

Immunohistochemical staining of liver and spleen tissues with CD3 confirmed the presence of Jurkat/CAR T-cells in the liver and spleen, in contrast with the control tissue from mice not injected with Jurkat/CAR T-cells (Fig 8A). Immunohistochemical staining of Raji and K562 tumors of the day 3 group with CD3 showed barely-visible stained cells in the periphery of the tumors. In the day 7 group, hPBMC CAR T-cells showed proliferation throughout Raji tumors; however, Jurkat/CAR T-cells were not visualized (Fig 8B). No K562 tumors were stained with CD3 antibody.

## Discussion

In this study, *in vivo* CAR T-cell trafficking was feasible for seven days after intravenous injection by ^89^Zr-DFO labeling and PET/MR images. The CAR T-cells initially reached the lungs and gradually migrated to the liver by day 1, where they remained for the rest of the experimental period. Migration to the spleen was also evident and showed high SUV on PET/MR images, although %ID was relatively low, and the spleen is small in immunocompromised NSG mice. The organ distribution of ^89^Zr-DFO-labeled cells, quantitatively assessed by PET/MR images, was confirmed with an *ex vivo* biodistribution study in which we analyzed the radioactivity of each organ harvested from the mice on day 7. This pattern of distribution was observed in both ^89^Zr-DFO labeling of Jurkat/CAR T-cells and CAR T-cells from human peripheral blood. Although we did not perform control *in vivo* imaging, biodistribution of possible free form of radiotracer, such as ^89^Zr-DFO or ^89^Zr-chloride, is different to that of ^89^Zr-DFO labelled CAR T-cells shown in this study. The distribution of cells after injection of unlabeled Jurkat/CAR T-cells was also confirmed by flow cytometry, Alu PCR, and IHC with isolated tissues from the sacrificed mice, on day 3. We could reliably and noninvasively track the distribution after cell administration using ^89^Zr-DFO labeling of CAR T-cells and PET/MR imaging, as suggested by FDA guidance. In quantitative analysis of organ distribution of cell populations, previous studies with radioisotope labeling had a lack of specific organ distribution data because of the difficulties in gathering anatomical information with PET imaging alone. The current study was performed using a hybrid imaging system. Here PET and MR imaging allowed organ-specific detection of the cell signal, together with structural information. The quantitative nature of PET imaging allows for longitudinal studies that provide information on relative levels of CAR T-cells at both the site of disease and potential off-target sites of accumulation. Monitoring the locations and potential secondary sites involved in CAR T-cell trafficking enables us to characterize the activity of administered cells and the safety profile.

^89^Zr-DFO was used for labeling CAR T-cells in this study, instead of ^89^Zr-oxine complex, which was used by Weist et al. [19], because it has more stable covalent binding between the DFO and cell surface protein [16]. With ^89^Zr-DFO labeling of human immune cells, radioactivity concentrations of radioisotope-labeled cells of up to 0.5 MBq/10^6^ cells were executed without an unfavorable effect on cellular viability, and cell efflux studies showed high radiolabel stability, with virtually no loss of tracer, for up to seven days [16]. Whereas a significant amount of ^89^Zr-oxine can efflux from various kinds of cell, there is potential uptake of a small amount of free ^89^Zr released from cells into the bones or kidneys, as shown in the report by Weist et al. [19]. In our PET images of ^89^Zr-DFO-labeled cells, less radioactivity accumulated in the bones and kidneys until seven days after injection, compared with the results reported using ^89^Zr-oxine labeled cells. Furthermore, decay corrected injected activity was maintained on serial PET images (98.9 ± 8.3%), without significant loss of activity from the body. These findings suggest stable cell labeling by ^89^Zr-DFO *in vivo*, as demonstrated in other studies.

The cell viability and functionality, such as cytokine production, were not affected by labeling CAR T-cells with ^89^Zr-DFO at radioactivity cell concentration of 98 kBq/10^6^ cells. However, proliferation ability was slightly decreased over the next several days compared with that of unlabeled cells. It is important to consider the effect of the radiation dose on cells of hematopoietic origin, which are radiation sensitive. In particular, ^89^Zr has high-energy gamma emissions of 908.97 keV, which may limit the radioactive dose; furthermore, the radiolabeling procedure is potentially cytotoxic. Therefore, we reduced the dose of ^89^Zr-DFO, when labeling CAR T-cells from hPBMC, to 74 kBq/10^6^ cells, compared with 185 kBq/10^6^ cells in the labeling of Jurkat/CAR, after careful dose optimization because of hPBMC sensitivity.

The distribution pattern of CAR T-cells in this study was similar to that of previous studies. Following intravenous administration, human T-cells migrated in a manner similar to that reported in humans, but penetrated poorly into established tumors. Following intravenous administration, human T-cells initially reached the lungs, where they remained for more than four hours. After that, the T-cells redistributed to the liver, spleen, and lymph nodes [20]. This pattern was seen using all T-cell populations tested, regardless of tumor status or transgene cargo, and closely mimicked patterns of migration seen in human (via infusion) [21–23] or murine T-cell [24,25] recipients. According to Charoenphun et al., ^89^Zr-oxine labeled myeloma cells were injected intravenously and were found within the lungs at 30 minutes after injection, but migrated to the liver and spleen on day 1. This distribution continued until day 7 [26]. Similar distributions of ^89^Zr -oxine labeled dendritic cells were observed by Sato et al. [18]. Weist et al. also found that the highest CAR T-cell activity was in the spleen, followed by the liver [19]. In these studies, lung activity was significantly lower on day 7 than the activity in the liver or spleen. In contrast, Bansal et al. found that mesenchymal stem cells exhibited persistently high activity in lung images, up to day 7, and their biodistribution study also showed the highest activity in the lung (approximately 50%ID), followed by the liver (approximately 25%ID) [16]. It has been suggested that the slow migration of transferred T-cells through the lungs may be due to low pulmonary circulatory pressure, coupled with the narrowing of capillaries during expiration [23]. Importantly, activated T-cells cross the pulmonary circulation with reduced intravascular velocity, compared with that of their inactivated or naive counterparts [27,28]. This delay may, in part reflect an enhanced interaction between high-affinity state LFA-1 (T-cells) and ICAM-1 (endothelium) [27]. Delayed clearance of activated T-cells during their first pass through the lungs may be highly related with pulmonary toxicity that can occur following infusion of CAR T-cells. In one published incident, fatal adult respiratory distress syndrome occurred rapidly following infusion of > 10^10^ T-cells, targeted against ErbB2 using a trastuzumab scFv coupled to a fused CD28/4-1BB/CD3ζ endoplasmic domain [29].

In this study, T-cell migration to the target tumor was not observed on PET images, unlike in the reports by Sato et al. and Weist et al. It was disappointing to observe that only a minority of intravenously administered CAR T-cells migrated to tumor deposits, even though we used CD19 CAR T-cells with proven efficacy in animals and humans. There are several potential causes of this phenomenon. We used Jurkat/CAR T-cells in this study, which are leukemia cells and not true T-cells. Although Jurkat cells were used for convenience in this experiment, their actual biological behavior may be different from normal T-cells. Second, the solid subcutaneous tumor xenograft model used in this study was a different tumor environment from that of human acute lymphocytic leukemia of the blood, for which CD19-CAR T-cells have been shown to have a dramatic therapeutic effect. Also, the differences in the antigen-presenting status of the tumor, tumor microenvironment etc. between the xenograft model generated from Raji cells and those of previous studies by Sato et al. (B16 murine melanoma cells [18]) and Weist et al. (prostate cancer PC3-overexpressed cells [19]) may have affected the accumulation of CAR-T-cells within tumor tissue [30]. Third, detection of cell trafficking by imaging has important limitations. Cell trafficking may not be detectable via PET imaging when small amounts of T-cells, below detectable limits, are injected. Furthermore, after homing to the tumor, activated CAR T-cells can proliferate and dilute the labeling signal. In our IHC & PET imaging data, hPBMC CAR T-cells showed proliferation within Raji tumor tissues after CD19 targeting (Fig 8B); however, there are rare PET imaging signals in tumors.

There are several limitations to this study. First, we did not show a therapeutic effect for CAR T-cells because our experiments were conducted mainly with Jurkat/CAR T-cells instead of hPBMC CAR T-cells. Limited imaging experiments were possible using hPBMC CAR T-cells and only two mice. Second, the biodistribution study was only performed using direct cell labeling strategies. The direct cell labeling method cannot visualize cell proliferation after homing to the target tumor [31]. Third, we did not perform a control *in vivo* distribution study with ^89^Zr-DFO or free form of ^89^Zr, since this study was to explore the feasibility and we tried to use a minimum number of mice. To date, several previous *in vivo* biodistribution imaging studies have been well performed and are comparable with ours [15], in which the biodistribution patterns of various forms of ^89^Zr, including ^89^Zr-DFO, were very different from those of the ^89^Zr-DFO labelled cells. Therefore, we think that the PET/MR image results in this study reflect the biodistribution of the ^89^Zr-DFO labelled CAR T-cells.

In this study, ^89^Zr-DFO labeling CAR T-cell direct imaging had great advantages in enabling observation of the initial behavior of the injected cells, and in the whole-body distribution in real-time, with very low background activities, which, in principle, are not present in other host tissues or cells. This direct labeling did not involve genetic manipulation of the therapeutic cells and, therefore, it was a simple way to trace initial injected CAR T-cells.

Further studies that use both direct and indirect labeling strategies, where reporter genes are inserted in the vector, should be carried out using hPBMC CAR T-cells.

## Conclusions

With ^89^Zr-DFO labeling of CAR T-cell, real-time *in vivo* cell trafficking was feasible through PET imaging, after administration of these cells to the body. Thus, ^89^Zr-DFO-labeled CAR T-cell PET imaging can be used to investigate the cell kinetics, *in vivo* cell biodistribution, and safety profile of CAR T-cell therapies that are developed.

## Supporting information

S1 File Original data of the present study. (XLSX)

S2 File Flow cytometry analysis for CD19 expression of Raji and K562 cell lines. (PNG)
