# Supplementary figures and images for: Feasibility of real-time in vivo 89Zr-DFO-labeled CAR T-cell trafficking using PET imaging
Source: PLoS One. 2020 Jan 7;15(1):e0223814. doi: 10.1371/journal.pone.0223814 (PMC6946129; doi:10.1371/journal.pone.0223814)

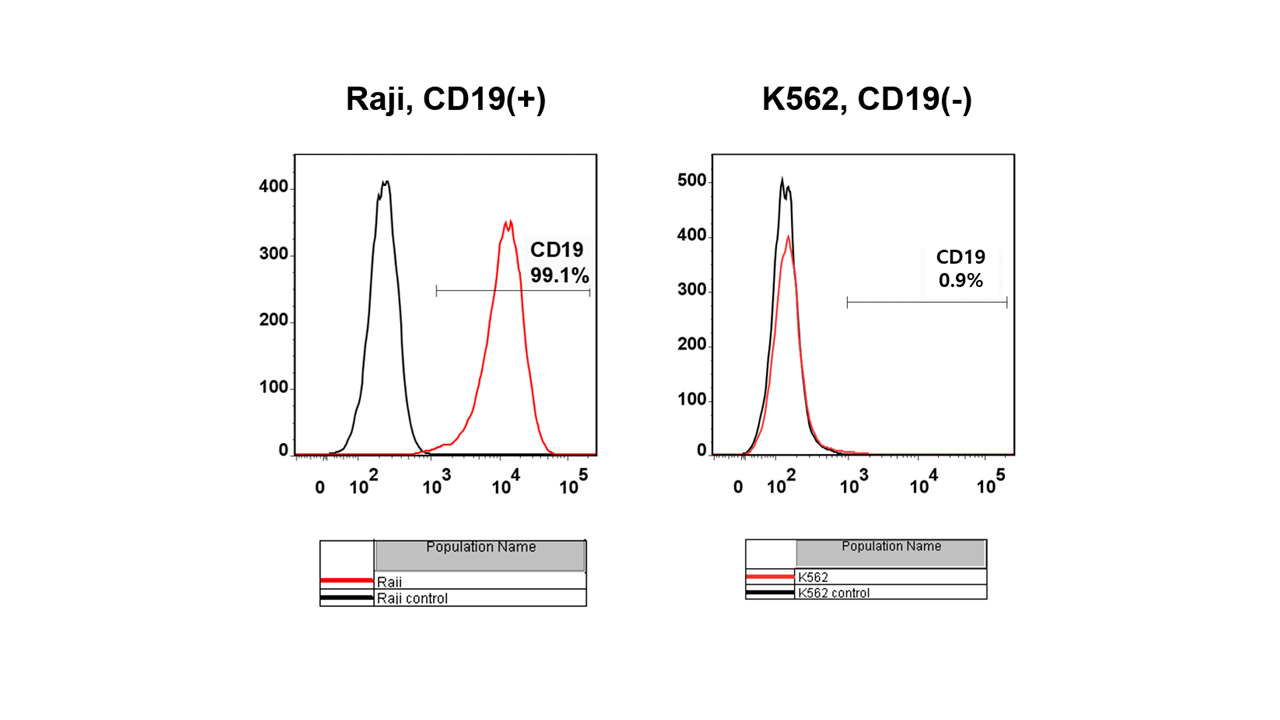

Supplement: S2 File — (PNG) [file pone.0223814.s002.PNG]
